# Roundtable discussion on the Third Global Symposium on Health Systems Research: why prioritise talk over aid in the midst of the Ebola crisis?

**DOI:** 10.1186/s12913-015-0842-z

**Published:** 2015-05-07

**Authors:** Jeffrey V Lazarus, Dina Balabanova, Kelly Safreed-Harmon, Karen Daniels, Kopano Matlwa Mabaso, Martin McKee, Tolib Mirzoev, Adnan A Hyder, Sofia Gruskin

**Affiliations:** CHIP, Centre for Health and Infectious Disease Research and WHO Collaborating Centre on HIV and Viral Hepatitis, Rigshospitalet, University of Copenhagen, Copenhagen, Denmark; Department of Global Health and Development, London School of Hygiene and Tropical Medicine, London, UK; Health Systems Research Unit, South African Medical Research Council, Cape Town, South Africa; Nuffield Department of Population Health, University of Oxford, Oxford, UK; The Centre for Health and Social Change (ECOHOST), London School of Hygiene and Tropical Medicine, London, UK; Nuffield Centre for International Health and Development, Leeds Institute of Health Sciences, University of Leeds, Leeds, UK; Department of International Health, Johns Hopkins Bloomberg School of Public Health, and Johns Hopkins Berman Institute of Bioethics, Baltimore, MD USA; Program on Global Health and Human Rights, Institute for Global Health, Gould School of Law, Keck School of Medicine, University of Southern California, Los Angeles, CA USA; Harvard School of Public Health, Harvard University, Boston, MA USA

## Abstract

Health systems experts from around the world discuss why they were meeting at the Third Global Symposium on Health Systems Research while people were dying of Ebola in West Africa.

Why were we meeting while real people were dying of Ebola in West Africa? This is what Christian Mpoyi Kalala, owner of a restaurant in Cape Town, asked one of the authors of this roundtable article. All of the money spent for smartly dressed people to travel to Cape Town, and stay in comfortable accommodations, and meet for days at a fancy convention centre – why not use the money instead to buy medicines for people with Ebola?

Those of us who had the good fortune to participate in the Third Global Symposium on Health Systems Research in late 2014 know very well that the event enabled us to share knowledge in ways that might potentially accelerate health systems strengthening efforts worldwide. Such work is vital because the global health community cannot afford to lurch from one headline crisis to another, whether the crisis unfolds across years and decades, as the AIDS epidemic has, or whether it is driven by a disease that can overwhelm entire countries in months.

The research, policy and advocacy efforts nurtured by the Third Global Symposium will surely demonstrate its worth to the global health community in many ways in the coming years, and there is a clear demand for more of these types of fora at the global and regional levels. The purpose of this roundtable discussion is not to invite health systems experts to argue the case for why such gatherings are warranted. Instead, we would like to invite some of those who attended the Cape Town event to take up the challenge of explaining to Mpoyi Kalala and other members of the general public why our efforts are needed.

We anticipate that this journal article will be read primarily by health policy and health systems specialists, and yet we suggest that our approach to the article presents a valuable opportunity for roundtable contributors and readers alike. It is not enough for us to have a discourse among ourselves about what needs to happen in order for health systems to be better prepared for the next infectious disease epidemic or outbreak. It is not even enough for those in health systems circles to communicate in ways that bring other actors such as communicable and non-communicable disease experts into their circles. We are leaving out the most important stakeholders of all if we are unable to explain the value of our work to the broad general public.

With this assertion as the premise for the following roundtable discussion, we have invited a number of people who attended the Third Global Symposium to make brief contributions answering the following simple question: how do you respond to Christian Mpoyi Kalala?

In other words, why – in layperson’s terms – should efforts to share knowledge about a broad array of health systems issues be prioritised at a time when resources are urgently needed to address an acute health crisis?

## Neglected health systems: a river running dry

By **Karen Daniels**, Specialist Scientist, South African Medical Research Council

The current Ebola crisis in West Africa is extremely troubling and should never have been allowed to occur. As an international public health community we have failed the people of West Africa by not responding immediately when first alerted to the outbreak. Global health leaders failed by not taking action, and we as researchers and activists failed by not being vigilant of the impending crisis and not putting pressure on these leaders of our community. But our greatest failure is that Ebola has been known to us for nearly 40 years and yet we failed to put pressure on the pharmaceutical industry to develop a cure. Until now Ebola has been a disease experienced only by a few, poor, rural people, and thus there is no market incentive for developing a cure. But why indeed is the health industry to be largely driven by market incentives and not human rights imperatives? For these our failures, we must hang our heads in shame.

In acknowledging and examining our failures, the act of coming together at a global symposium rather than being at the frontline is not diverting energy from finding a solution to this crisis. While the crisis is immediate, the problem is symptomatic of the long-term neglect of the health system. Thus addressing it requires both immediate action (which I agree needs to be intensified) as well as analysis of how the health systems of countries in West Africa collapsed and how these health systems might now enter into a process of restoration. Our symposium addressed the longer-term issues.

A health system can be thought of as an underground river, feeding the delivery of adequate and appropriate health care above ground. There are many components to this system which can be thought of as the nutrients: financing, information systems, supply mechanisms, human resources, physical infrastructure, appropriate training, governance, and so on. But right now the underground river is nearly dry. Even if we magically had large supplies of an effective treatment and an effective vaccine at our immediate disposal, the lack of capacity to deliver these goods means we would still fail.

The Cape Town symposium enabled new and experienced researchers, policy-makers and implementers to learn together about how to fix broken health systems. We focused on a combination of historical experience and new innovations in our efforts not only to respond to this crisis, but to avert future crises.

## The Cape Town symposium: a force for holding us accountable to each other

By **Kopano Matlwa Mabaso**, University of Oxford

The health systems conference is an opportunity for us, all of us, health care workers, community representatives, politicians, academics, business people and any other interested parties, to come together and share ideas on how we can tackle large, monstrous problems in a decisive and definitive way. We, the conference participants, are “real people” too, real people who care, who want to make a difference, who want to make things better.

The Ebola crisis we see today is a consequence of decades of devastation in the region. And so throwing the conference budget at the problem is unlikely to result in sustained change; we will only manage to buy ourselves a little bit of time, until we are faced with the next crisis.

All of that said, sometimes we (the research community) are part of the problem. Sometimes we allow our own career ambitions, our prejudices, our greed, to get in the way of the real work we should be doing. Sometimes we sit in our offices and think we are making a difference with our research and policies, when really we are making short-sighted decisions that are doing more harm than good.

So the conference serves another purpose: It is an opportunity to bring us all back together, from our various backgrounds and various disciplines, on the same platform, to remind each other of our common humanity and of the urgency with which social injustices need to be addressed. *And* to reprimand each other when we act in contradiction to these shared values.

It is said, *“If you want to go fast, go alone. If you want to go far, go together”.*

We want to go far. The conference is one of multiple vehicles for us to go far, together.

## The Ebola epidemic is a collective failure

By **Martin McKee,** Professor of European Public Health, London School of Hygiene and Tropical Medicine

The response to the current epidemic of Ebola could make one wonder if the Enlightenment had ever happened. An American volunteer nurse who returned from West Africa was detained at Newark airport and treated like a criminal. Even after being tested and found to be Ebola-free, she faced legal attempts to confine her to her house in the state of Maine. Some high-income countries have introduced airport screening even though there is no evidence that it is effective. West African women have responded to the stigma they face simply on grounds of their nationality by posting pictures of themselves on the internet holding signs saying, “I am a Liberian, not a virus”. Another social media response mocks the pervasive ignorance about Ebola with a map that depicts the countries of Guinea, Liberia and Sierra Leone with the caption, “Ebola”. All other African countries appear in another colour with the caption, “No Ebola”.

This is not the fourteenth century, when those witnessing the effects of the Black Death could be forgiven for seeing it as punishment by God. We know a great deal about the Ebola virus, its transmissibility and its epidemiology. The challenge is to translate that knowledge into policy and practice.

Policy failings on Ebola go beyond the practical management of those perceived, rightly or wrongly, to be at risk. Even though the virus was identified almost 40 years ago, neither a vaccine or a treatment is currently available. This is inexcusable. The Ebola virus is not like the constantly changing influenza virus. Nor is it like HIV, attacking and infecting the immune system, making vaccine development very difficult. The problem is not a technical one. It is a matter of the failure of the existing market-based system of drug discovery and development to produce drugs whose beneficiaries are few and poor. Although this is a wider problem, apparent in the lack of investment in new antibiotics, Ebola has brought it to the fore.

Ebola has also cast light on the abysmal failure, over decades, to invest in health systems. It is no coincidence that it has emerged in some of the countries with the weakest and worst-resourced health systems in Africa. These problems could and should have been addressed long ago. But they were not. One reason is a lack of political commitment. Another is the uncertainty about what needs to be done, in policy and practice, to achieve real change.

Conferences such as that in Cape Town have a crucial role to play, making visible the scale and nature of the problems that exist, sharing ideas and experiences of what may work, and bringing together the global community of health researchers and policy-makers who return to their countries with renewed commitment to making this world a safer and healthier place.

## How do we motivate health workers to work in dangerous areas?

By **Tolib Mirzoev,** Senior Researcher, University of Leeds

In the context of health systems, the different systems components need to work together optimally to ensure that often-scarce resources are not wasted. Health workers such as nurses, doctors and volunteers constitute one example of a systems component. Another example is infrastructure, including ambulances and clinics where sick people can be treated. The Cape Town symposium sought to improve our understanding of how these systems components shape responses to health challenges in different countries.

Discussions during the symposium conveyed the message that while interactions between health systems components are complex, each component itself is also complex. One session asked: *what motivates health workers to provide high-quality healthcare?* Participants made the point that apart from staff recruitment and training, the adequate and timely presence of motivated staff is particularly important in areas affected by epidemics such as Ebola. This is because prompt diagnosis, treatment and sometimes even quarantine of infected individuals are essential to prevent the disease from spreading further [1]. The lack of staff motivated to work at the community level can greatly undermine such efforts.

Different countries are currently sending health volunteers to West Africa to help stop the Ebola epidemic. This aid is no doubt valuable in the short term. However, the reality is that the affected countries’ own health workers will ultimately bear the burden of containing any further cases in the aftermath of international relief efforts. Furthermore, health workers themselves are also people – they need compelling reasons to provide healthcare to their patients and communities while also looking after their own families and social networks.

In one Cape Town session we learned − from experiences in Uganda [2] and Bangladesh [3] – about different ways of motivating health workers to perform jobs in remote and dangerous areas. Money, although important, is not always the only answer. Safety and security, infrastructure to support workers and their families (such as schools for children and land to grow food) and opportunities for professional development (such as training) were found to be equally important.

When considering the justification for having this symposium while Ebola continued to devastate many communities, we need to recognise that making resources available is only part of the response required to manage Ebola and other health crises. We need to ask how these resources can best be used. Identifying and applying effective strategies to motivate health workers to serve under difficult circumstances is one of the many aspects of successful health systems, and the different discussions at the Cape Town symposium promoted a better understanding of these complex issues.

## Act now or reflect first in response to health systems challenges? Ethical considerations

By **Adnan A. Hyder**, Professor and Director, Health Systems Program, Department of International Health, Johns Hopkins Bloomberg School of Public Health

Health systems around the world should provide benefits to people – an ethical principle known as *beneficence*. According to this principle, securing the health of people should always be the main focus of efforts to address a health situation such as the Ebola crisis. And yet we find that health systems charged with responding to urgent needs often lack essential human resources, medical supplies and key infrastructure. Even if some of what is missing can be delivered quickly by external agents, these health systems do not have the capacity to absorb a sudden influx of resources efficiently. When health systems remain under-resourced for long periods of time, they are at risk of delivering low-quality and inefficient health care to people; thus not being *responsive* to the health needs of their populations (another principle). Responding to this concern, global health actors and health systems experts have promoted strategies for improving health systems through sustained investments and for translating these investments into effective services.

Serious health problems confront nations every day. Yet some problems unfold more rapidly with more severe consequences than others, such as floods in Pakistan, famine in Sudan, or Ebola in West Africa. It is at these times that the tension between immediate action and focused exploration of longer-term options becomes acute. The question is: do we act now or do we gather – as at the Third Global Symposium – to share knowledge and deliberate about how the available evidence should inform current and future actions?

Those who favour an urgent response are correct that immediate measures are often needed to limit the death and disability resulting from adverse events. Those who support more research and discussion are also right – the evidence base regarding what works needs to be strengthened if we are to improve our response. But these two options are not as disconnected as some might think. People who rush to help in situations such as the current Ebola outbreak are actually making decisions on the basis of evidence from previous experiences. This is true each time we help an *individual*, such as when physicians treat a patient, and it is also true when we support an *institution* or a *system*, such as when we develop a financing model or improve quality of care. Thus “now or later” is a false dichotomy.

At the core of this discourse on health system actions (such as those recently spotlighted at the Third Global Symposium) is concern for the fundamental ethical principle of *social justice*; it is the reason for many health system actions to improve health or distribute it equitably among all people. It is therefore critical that we respond to health threats with a combination of immediate relief and long-term strategies that enable national health systems to be better prepared for the future.

## Talking the hard issues: why health systems research matters for sexual and reproductive health and rights

By **Sofia Gruskin**, Director, Program on Global Health and Human Rights, Institute for Global Health, University of Southern California; and Adjunct Professor of Global Health, Harvard School of Public Health

I am by nature sympathetic to any repudiation of global talk shops, even without a looming global Ebola crisis, and must admit to getting off the plane in Cape Town with a critique already in mind. How often should we once again listen to one another? Those of us who travel these circuits seeing each other in Montreux, Beijing and Cape Town, do we really have something to learn from one another which we cannot get by simply taking the time to stay current with the literature?

Ebola has shed light on failing health systems in the range of countries most impacted, but these failings are not only relevant in times of crisis. Highly vulnerable populations are highly vulnerable to disease and ill-health even when the eyes of the world are not watching, and most especially when the conditions affecting them bring to the fore issues of social values, religion and morality. More than in many health systems research areas, sexual and reproductive health and rights generate strong opinions often with insufficient attention to evidence rather than ideology. There has been limited capacity to produce research that will help meet current as well as post-2015 goals and commitments relating to sexual and reproductive health in ways that advance equity and rights. Donor interest is already scant, and training curricula and other efforts inadequate to prepare the next generation of students and practitioners.

In the global debates happening right now, there is a tug of war between those who recognise the importance of sexual and reproductive health and rights for the achievement of social justice and those who do not. Of relevance to how the gender and rights issues embedded in the Ebola response will be addressed going forward, increased conservatism, lack of political will, and to some degree, outright resistance to research and training in sexual and reproductive health and rights have become central challenges. Finding ways to work within politically and socially constrained contexts – and, when necessary, to contest them – is a key priority. Figuring out how best to do this cannot happen simply through reading or internet exchanges.

The Third Global Symposium included close to a dozen panels addressing rights, gender and social exclusion, areas as relevant to Ebola as to sexual and reproductive health. I was much more likely to follow these sessions than the ones on insurance schemes and economic reform, yet the design of the symposium encouraged far more crossover than usually occurs at such events. I made my way home convinced that this had been more than an expensive opportunity to see friends. The relatively small size of the gathering and most importantly the cross-section of people in attendance had stimulated thought and presented opportunities to channel resources to important projects. Furthermore, in this increasingly conservative time, some of what occurred might actually help sway the global health architecture to more fully address not only Ebola and other immediate crises but the hot-button and uncomfortable issues that are central to advancing rights and health into the next millennium.
